# Comparison of Modular Control during Smash Shot between Advanced and Beginner Badminton Players

**DOI:** 10.1155/2018/6592357

**Published:** 2018-06-13

**Authors:** Naoto Matsunaga, Koji Kaneoka

**Affiliations:** ^1^General Education Core Curriculum Division, Seigakuin University, 1-1 Tosaki, Ageo, 362-8585 Saitama, Japan; ^2^Waseda Institute for Sport Sciences, Waseda University, 2-579-15 Mikajima, Tokorozawa, 359-1192 Saitama, Japan; ^3^Faculty of Sport Sciences, Waseda University, 2-579-15 Mikajima, Tokorozawa, 359-1192 Saitama, Japan

## Abstract

This study investigated muscle synergy during smash shot in badminton and compared synergies of advanced players (more than 7 years experience) and beginner players (less than 3 years experience). The dominant hand of all players was the right side. Muscle activities were recorded on both sides of the rectus abdominis, external oblique (EO), internal oblique/transversus abdominis (IO/TrA), and erector spinae. Additionally, the right side of the biceps brachii, triceps brachii, flexor carpi radialis, flexor carpi ulnaris, and flexor digitorum profundus muscle activities were recorded. All data was obtained using surface electromyography. Synergy was extracted from electromyography signals using nonnegative matrix factorization. Extracted synergies in each group were compared using scalar product (SP) which is the similarity index. As a result, two synergies were extracted in the beginner players and three synergies were extracted in advanced players. Beginner and advanced players had one synergy in common (SP = 0.86) that was mainly on the left side of the EO. It activated in the early stroke and had a role of side bending from the left to hit the shuttlecock at a higher point. Another synergy that had coactivation of the IO/TrA and forearm muscles at impact was extracted only for advanced players and it may enhance the smash shot performance in badminton.

## 1. Introduction

Bernstein proposed the “module hypothesis,” and recently, based on this concept, a neural control mechanism during locomotion was investigated through electromyography (EMG) using nonnegative matrix factorization (NMF) [1]. From NMF, EMG is divided into muscle synergy and an activation coefficient. Muscle synergy indicates the muscle combination to be mobilized and activation coefficient indicates the activation timing of muscle synergy. The modules include these factors and human movements are performed by the combination of several modules. Recently, muscle synergy analysis has been applied to sports activities and several studies reported the synergy while running, cycling, rowing, and swimming [2–6]. Vaz et al. compared the synergy during breaststroke between elite and beginner swimmers and the timing of propelling the water of beginner swimmers was earlier than elite swimmers [6]. Sawers et al. reported that the number of synergies of elite dancers during walking was lots than that of beginners [7]. From these reports, synergy is different due to competitive level and beginners may improve if they get the synergy as elite athletes. Therefore, it is considered that to clarify the difference of synergy due to competitive level is important information for athletes and coaches.

The Badminton World Federation (BWF) estimated that about 200 million people of all ages and levels play the game worldwide [8, 9]. Badminton is a highly dynamic sport with the shuttlecock being struck at over 300 km/h. The BWF coaches' manual states that players are required to have a high level of motor coordination [9]. However, previous studies that targeted sports activity did not include ball games because the motion of player is influenced by the motion of the opponent player or by the return ball by the opponent player [2–6]. Although we could not integrate the player's motion exactly as in ball games, we thought that it was important to assess the muscle coordination pattern for the improvement of competitiveness. Thus, the purpose of this study was to investigate whether muscle synergy during overhead stroke in badminton depends on the competition level. If we could clarify the factor to improve the performance using synergy analysis in this study, synergy analysis may help not only badminton but also any other ball game the plan of training program to improve the performance.

## 2. Materials and Methods

### 2.1. Subjects

Participants in this study included seven advanced players who had been playing badminton for more than 7 years (age: 20 ± 1 years, height: 169.7 ± 5.7 cm, weight: 58.4 ± 4.9 kg, competition history: 8 ± 2 years) and six novice players who had been playing badminton for less than 3 years (age: 20 ± 2 years, height: 168.2 ± 9.1 cm, weight: 56.5 ± 10.0 kg, competition history: 2 ± 1 years). All subjects were men, all of them were right-hand dominant in holding the racket, and all played badminton two or three times per week at the recreational level. Exclusion criteria included a history of upper or lower limb and spinal disorder, neurological disorder, or lower limb and spine surgery. The Ethics Committee of our university (2013-033) approved this study and each subject provided informed consent to participate in this study. The experimentation was conducted in accordance with the Declaration of Helsinki (1964).

### 2.2. Instrumentation

The activities of the trunk and upper extremity muscles were measured using a wireless EMG telemeter system (BioLog DL-5000, S&ME Co., Japan) at 1000 Hz. Before the surface electrodes were attached, the skin was rubbed with a skin abrasive and alcohol to reduce skin impedance to a level below 2 k*Ω*. Pairs of disposable Ag/AgCl surface electrodes (BlueSensor N-00-S, METS Co., Japan) were placed on the rectus abdominis (RA), external oblique (EO), internal oblique/transversus abdominis (IO/TrA) [10], erector spinae (ES), biceps brachii (BB), triceps brachii (TB), flexor carpi radialis (FCR), flexor carpi ulnaris (FCU), and flexor digitorum profundus (FDP) muscles. Trunk muscle activity was measured on both sides and upper extremity activity was measured only on the dominant right side. Before any measurements were taken, the subjects performed maximum voluntary contraction (MVC) tests using manual resistance to normalize their EMG data. Simultaneously, video data was recorded at the side of the badminton court using a digital camera (EX-FH25, CASIO, Japan) at 240 Hz. The digital camera was synchronized with the EMG system using LED synchronizer (PH-105, DKH Co., Japan) which can conciliate the time of start of measurement between these systems.

### 2.3. Data Measurements

Subjects performed stretching, running, and practice badminton as a warmup for 20 min. After warmup, the EMG measuring equipment was setup. Then, muscle activity during a smash shot was measured using the following procedure. Subjects were asked to step into the starting position. When in position, the shuttlecock was launched toward the player and the player hit it. To keep a bare minimum the time lag of two measurement systems, we measured only one shot at one trial. This trial was performed until the subjects were successful thrice; therefore, the number of trials was different in each subject.

### 2.4. Data Analysis

**Figure 1:**
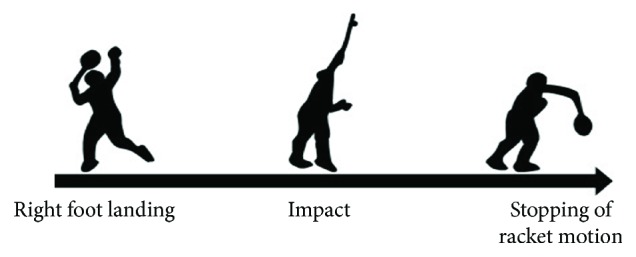
Phase dividing during badminton overhead stroke.

We defined the beginning of the stroke as the right foot landing and the finish of the stroke as the point of follow-through using video data (Figure 1). Then, MATLAB was used to analyze the EMG data. The raw data was band-pass filtered between 20–450 Hz and full-wave rectified. The EMG data was normalized relative to the MVC data. We extracted the stroke with the lowest noise in the EMG data from each trial. The data for each stroke selected was interpolated to 200 time points. Then, as previously described, NMF was performed to extract synergies based on the study by Lee and Seung such that

(1) $$\begin{array}{c} E=WC+e. \end{array}$$

In this equation, **E** is a *p*-by-*n* initial matrix (*p* = number of muscles and *n* = number of time points) that represents the normalized EMG matrix, **W** is a *p*-by-*s* matrix (*s* = number of synergies) that represents the muscle synergy, **C** is an *s*-by-*n* matrix that represents the activation coefficient, and **e** is a *p*-by-*n* matrix that is the residual error matrix [11].

To avoid local minima, the algorithm was repeated ten times for each stroke of each subject. Then, to finalize the number of synergies, we calculated the variance accounted for (VAF) [3, 12, 13]. Global VAF was calculated based on these studies such that

(2) $$\begin{array}{c} Global\;VAF=1-\frac{\sum_{i=1}^{p}\sum_{j=1}^{n}{\left(e_{i,j}\right)}^{2}}{\sum_{i=1}^{p}\sum_{j=1}^{n}{\left(E_{i,j}\right)}^{2}}. \end{array}$$

In this equation, *i* goes from 1 to *p* and *j* goes from 1 to *n*. We defined the number of synergies based on when global VAF exceeded 0.9 for the first time [3]. Additionally, local VAF was calculated based on Hug et al. [3] such that

(3) $$\begin{array}{c} Local\;VAF\;\left[m\right]=1-\frac{\sum_{j=1}^{n}{\left(e_{m,j}\right)}^{2}}{\sum_{j=1}^{n}{\left(E_{m,j}\right)}^{2}}. \end{array}$$

In this equation, *m* represents the muscle “m”. We defined the adoption standard that local VAF > 0.75 [14].

To compare **W** (muscle synergy) between advanced and novice players, the scalar product (SP) which is the similarity index between 2 synergies was calculated based on a study by Cheung et al. such that [15]

(4) $$\begin{array}{c} SP=\frac{\vec{W_{advanced}}\times \vec{W_{novice}}}{\left|\vec{W_{advanced}}\right|\left|\vec{W_{novice}}\right|},\;0≦SP≦1. \end{array}$$

We defined synergies as the same synergy when SP was greater than 0.75 [15]. Additionally, we evaluated the intrareliability and intragroup similarity using SP. We followed the methods of our previous study [16].

### 2.5. Statistical Analysis

To compare the number of synergies between groups, Mann–Whitney *U* test was performed using SPSS statistics 23 (IBM). The significance level was set at an alpha value of <0.05.

## 3. Results

**Figure 2:**
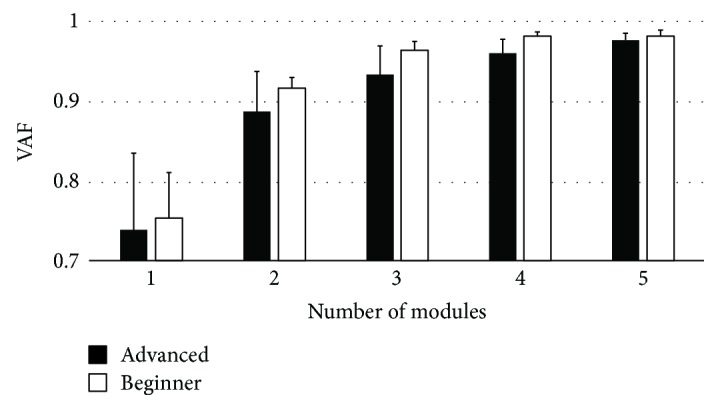
Relationship between the number of synergies and VAF. The number of synergies is decided when VAF exceeds 0.9 for the first time. VAF: variance accounted for.

Figure 2 shows the mean value of global VAF in advanced players and novice players, and Table 1 shows the local VAF when three synergies were extracted from advanced players and when two synergies were extracted from novice players. From these results, two synergies were extracted from the novice players (VAF: 0.92 ± 0.01) and three synergies were extracted from the advanced players (VAF: .93 ± 0.03). As a result of Mann–Whitney *U* test, the number of synergies was different between groups (*p* < 0.001).

Table 2 shows the mean value of SP for 3 trials in each subject. This result indicated that there was no problem for intrareliability in this study.

**Figure 3:**
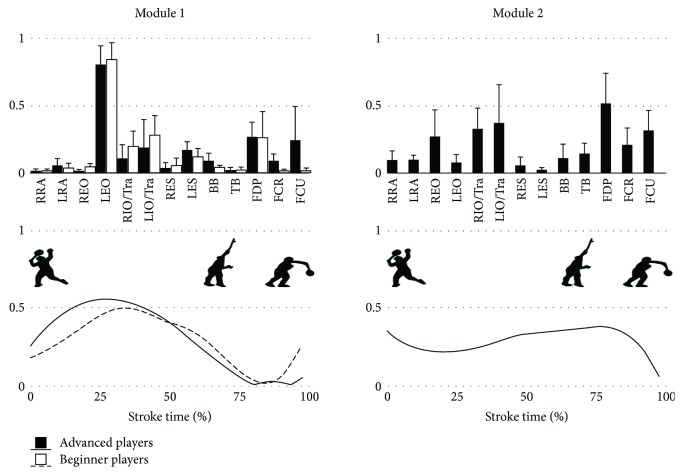
Extracted synergies during smash shot. RRA: right-side rectus abdominis; LRA: left-side rectus abdominis; REO: right-side external oblique; LEO: left-side external oblique; RIO/TrA: right-side internal oblique/transversus abdominis; LIO/TrA: left-side internal oblique/transversus abdominis; RES: right-side erector spinae; LES: left-side erector spinae; BB: biceps brachii; TB: triceps brachii; FDP: flexor digitorum profundus; FCR: flexor carpi radialis; FCU: flexor carpi ulnaris.

One of these synergies could not be grouped because the SP of intragroup was low due to the extreme individual differences. Therefore, one synergy for novice players and two synergies for advanced players were compared in this study. The SP of synergy 1 was 0.856 ± 0.067 and that of synergy 2 was 0.875 ± 0.044 in advanced players. Furthermore, the SP of synergy 1 in novice players was 0.890 ± 0.083. These results indicated that intragroup reliability was high.  Figure 3 shows the mean of each group of extracted modules. The SP that was used to indicate similarity of synergies was 0.86 for synergy 1 and therefore, this synergy was considered the same in both groups. Synergy 1 primarily engaged the left side of EO (LEO) and activated early in the stroke. Synergy 2 was only extracted for the advanced players and primarily engaged both sides of the IO/TrA and forearm muscles with peak activation at the time of impact.

## 4. Discussion

This study investigated the muscle synergy of 13 muscles groups during a smash shot by comparing advanced and novice badminton players. The main finding of this study was the cocontraction of the IO/TrA and forearm muscles at impact in advanced players.

Synergy 1 was considered the same in both groups. It primarily engaged the LEO muscles and peak activation was approximately 25–30% of overhead stroke. The EO muscle has the function of ipsilateral bending and contralateral rotation. Previous studies that analyzed the motion during a smash shot in badminton reported that the trunk bended for the nondominant side during the early stroke [17]. Although trunk motion was not analyzed in this study, it was assumed that the role of synergy 1 was to rotate the trunk and to bend the trunk in order for the nondominant side to hit a shuttlecock at a higher point.

Synergy 2 was only extracted for advanced players and focused on both sides of the IO/TrA and forearm muscles. The peak activation was about 80% of overhead stroke and corresponded to the impact of the shuttlecock. Badminton players need to regrip the handle of a racket depending on the position of shuttlecock and therefore coaches instruct that the grip on the racket should be relaxed. Additionally, the grip should be tightened at impact to hit a strong shot [9]. Therefore, this synergy indicates that advanced players gripped the handle of racket firmly before the impact point. Moreover, the contribution of the IO/TrA muscles was high in this synergy. The TrA activated as a postural control during upper limb movement [18]. Because the upper limb movement of the overhead stroke was rapid, the IO/TrA muscles needed high activation to control posture. Therefore, it is considered that the role of this synergy was to maintain the body balance to hit a strong shot.

The coaching for beginner become attached the style of overhead stroke therefore they perform repetitive practice swing. The badminton overhead stroke is similar to ball throwing form; therefore, they sometimes throw the shuttlecock. At that time, coaches instruct them to shake their arm like a whip therefore beginners end up being aware of the upper extremity (e.g., shoulder and forearm muscle) only. Thus, it is important to let them be conscious of the coordination of not only the forearm muscles but also the trunk muscles during practice in badminton coaching.

This study was performed as a preliminary study and therefore has some limitations. First, the sample size was small and included a synergy that could not be grouped in each player group. It is possible that the extracted individual synergies may have been influenced by the teaching method or career of the corresponding sports activity. Therefore, future study needs a large sample size. Second, it was possible that experiments were not conducted under the same conditions because the flight of the shuttlecock was not controlled. Despite of the situation, it is an important finding that both groups had the same synergy. This study was preliminary, and we can indicate the consideration when targeted ball games to perform the similar analysis for future study. Additionally, future work may need to analyze a practice stroke in addition to hitting the flying shuttlecock. Finally, adding motion analysis may clarify the difference in the number of synergies.

## 5. Conclusion

In this study, we compared muscle synergies during a smash shot between advance and novice badminton players using NMF. The number of synergies was different with three and two synergies extracted from advanced and novice players, respectively. One synergy was common between both groups. Another synergy was different for each subject due to the uncontrolled flying of the shuttlecock. An added synergy in advanced players indicates the coactivation of the forearm muscle and transversus abdominis at the impact.

## Figures and Tables

**Table 1:** Mean value of local VAF when advanced players had three synergies and novice players had two synergies. VAF: variance accounted for.

Muscle	VAF (mean ± SD)
Advanced players	
Right rectus abdominis	0.892 ± 0.021
Left rectus abdominis	0.851 ± 0.079
Right external oblique	0.946 ± 0.055
Left external oblique	0.938 ± 0.084
Right internal oblique/transverse abdominis	0.967 ± 0.042
Left internal oblique/transverse abdominis	0.972 ± 0.041
Right erector spinae	0.811 ± 0.078
Left erector spinae	0.857 ± 0.056
Biceps brachii	0.811 ± 0.079
Triceps brachii	0.852 ± 0.084
Flexor digitorum profundus	0.925 ± 0.075
Flexor carpi radialis	0.906 ± 0.067
Flexor carpi ulnaris	0.871 ± 0.071
Novice players	
Right rectus abdominis	0.857 ± 0.056
Left rectus abdominis	0.850 ± 0.032
Right external oblique	0.849 ± 0.082
Left external oblique	0.988 ± 0.014
Right internal oblique/transverse abdominis	0.985 ± 0.024
Left internal oblique/transverse abdominis	0.942 ± 0.049
Right erector spinae	0.863 ± 0.052
Left erector spinae	0.877 ± 0.060
Biceps brachii	0.854 ± 0.081
Triceps brachii	0.852 ± 0.065
Flexor digitorum profundus	0.964 ± 0.042
Flexor carpi radialis	0.913 ± 0.094
Flexor carpi ulnaris	0.902 ± 0.067

**Table 2:** Mean value of SP for 3 trials in each subject.

	Advanced players								Novice players					
		Subject 1	Subject 2	Subject 3	Subject 4	Subject 5	Subject 6	Subject 7	Subject 8	Subject 9	Subject 10	Subject 11	Subject 12	Subject 13
Synergy 1	Mean	0.865	0.933	0.955	0.944	0.935	0.873	0.939	0.938	0.951	0.889	0.886	0.867	0.982
	SD	0.078	0.077	0.043	0.042	0.006	0.004	0.013	0.039	0.035	0.068	0.073	0.106	0.012
Synergy 2	Mean	0.788	0.889	0.834	0.817	0.896	0.800	0.763						
	SD	0.036	0.016	0.065	0.050	0.034	0.050	0.010						

SP: scalar product.
